# Extracellular protein homeostasis: The dawning of a new age for human disease therapies?

**DOI:** 10.1002/ctm2.1305

**Published:** 2023-06-14

**Authors:** Mark R. Wilson, Sandeep Satapathy, Michele Vendruscolo

**Affiliations:** ^1^ School of Chemistry and Molecular Bioscience Molecular Horizons Research Institute University of Wollongong Wollongong New South Wales Australia; ^2^ Blavatnik Institute of Cell Biology Harvard Medical School Boston Massachusetts USA; ^3^ Centre for Misfolding Diseases Yusuf Hamied Department of Chemistry University of Cambridge Cambridge UK

1

**FIGURE 1:**
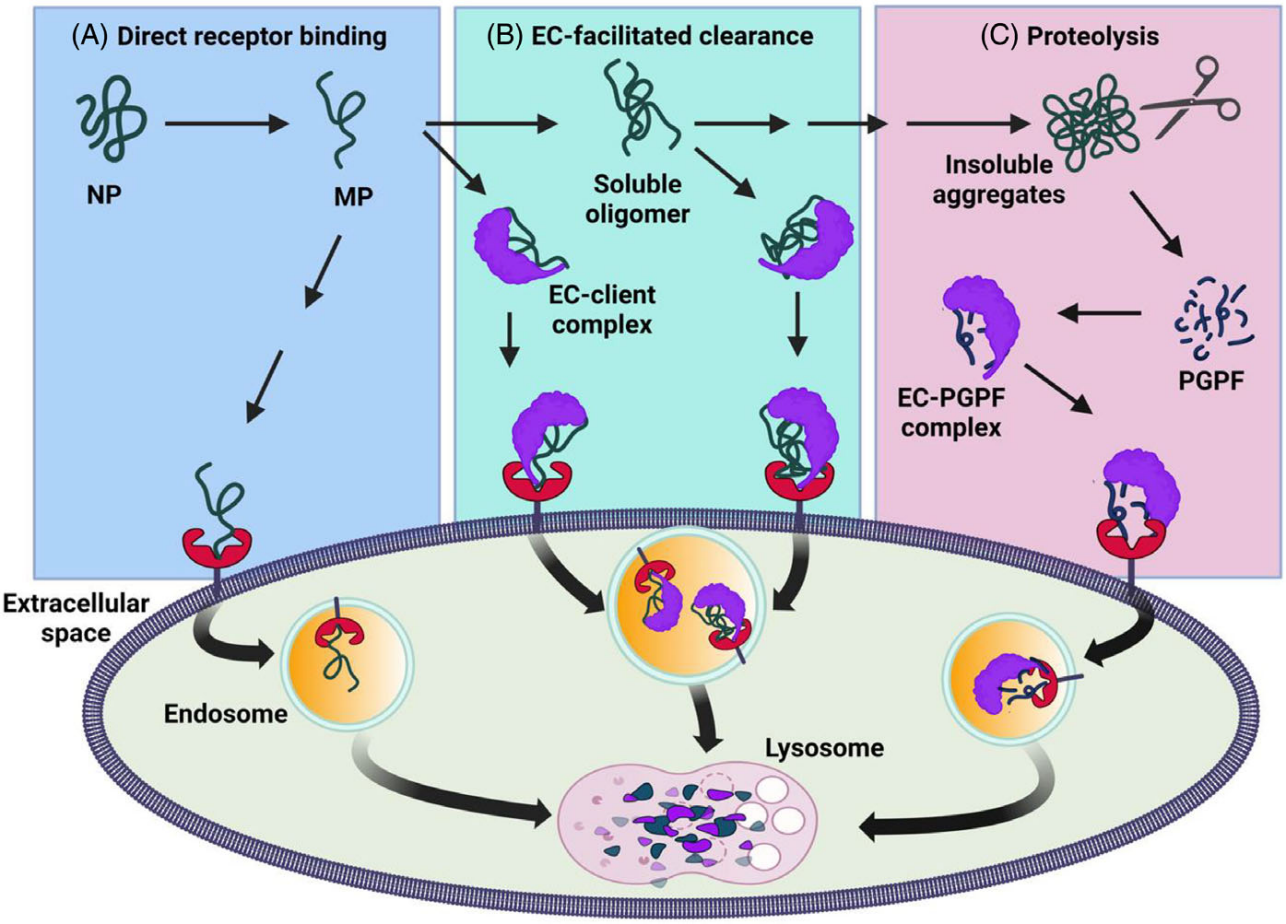
Overview of the extracellular protein homeostasis system. **(A** and **B)** As a result of physical and chemical stresses in the interstitial and cerebrospinal fluids, normally folded extracellular proteins (NP) can be converted to misfolded proteins (MP) which may **(A)** bind to cell surface receptors, or **(B)** form soluble complexes with extracellular chaperones (ECs) before doing so. **(C)** Large insoluble protein aggregates may activate extracellular proteolytic systems resulting in their digestion to small protease‐generated protein fragments (PGPFs), which in turn are bound by ECs. In all these situations, misfolded proteins (or resulting PGPFs) are taken up by cells via receptor‐mediated endocytosis and directed to lysosomes for degradation.

The protein homeostasis (proteostasis) system consists in a network of processes that governs the synthesis, folding, concentration, trafficking and degradation of proteins.^1^ The maintenance of protein homeostasis is especially important in extracellular spaces, where protein molecules are constantly exposed to oxidizing agents, large variations in pH and ionic strength, and mechanical and thermal stresses associated with fluid circulation (Figure 1). These environmental conditions can destabilize the native states (normal folded forms) of proteins, which can result in misfolding.^2^ In turn, protein misfolding may expose aggregation‐prone regions normally buried in the native protein interior, leading to self‐association into potentially cytotoxic aggregates. In Alzheimer's disease (AD), the amyloid‐β (Aβ) peptide aggregates into small oligomers that damage brain cells and later form insoluble fibrillar structures that deposit into large amyloid plaques.^3^ Extracellular protein homeostasis is tasked with preventing disease by controlling the process of protein misfolding in extracellular fluids, safeguarding against toxic protein aggregates that may arise, and mediating their systematic clearance. This perspective article provides a therapy‐focussed commentary to extend a discussion presented in a recent review of extracellular protein homeostasis in neurodegenerative diseases.^4^

Extracellular chaperones (ECs) are central components of the extracellular protein homeostasis system (Figure 1). ECs are secreted proteins, abundant in body fluids, that specifically interact with extracellular misfolding proteins to inhibit their aggregation, neutralise their toxicity, and facilitate their receptor‐mediated endocytosis and subsequent degradation in lysosomes. By virtue of these actions, ECs are emerging as key players in processes that operate to protect the human body from a variety of disease pathologies causally associated with the excessive misfolding and aggregation of specific extracellular proteins (Table 1). The extracellular protein homeostasis network may also include proteolytic systems, which could assist in the clearance of larger extracellular protein deposits (Figure 1).

**TABLE 1:** Examples of human diseases associated with extracellular protein misfolding and aggregation.^5^

Disease	Aggregating protein or peptide
Alzheimer's disease	Amyloid β peptide (Aβ)
Prion disease	Prion protein
Type II diabetes	Amylin (or IAPP)
Atherosclerosis	Apo‐B100
Hemodialysis‐related amyloidosis	β2‐microglobulin
Transthyretin amyloidosis	Transthyretin
Systemic AL amyloidosis	Immunoglobulin light chain
Systemic AA amyloidosis	Serum amyloid‐A protein (SAA)
Corneal dystrophy	Keratoepithelin
British and Danish familial dementias	ABri and ADan peptides
Lysozyme amyloidosis	Lysozyme
Vascular dementia	Medin

Abbreviations: AL, amyloid light chain amyloidosis; AA, amyloid A amyloidosis.

Since the discovery of clusterin (CLU) as the first known mammalian EC just over 20 years ago, about 20 more ECs have been identified.^6^ Each of these ECs offers the potential to be developed as therapeutic agents to treat the types of human diseases shown in Table 1. In this discussion, we will focus on CLU to illustrate possible translational pathways that other ECs may follow in the future.

Like many of the other known ECs, CLU is multifunctional. In addition to its potent chaperone activity and demonstrated roles in the clearance of Aβ and apoptotic cells,^7^, ^8^, ^9^ CLU is also an inhibitor of the terminal complement pathway^10^ and matrix metalloproteases.^11^ This broad range of activities suggests that CLU is likely to exert pleiotropic anti‐inflammatory effects in vivo, which have the potential to be harnessed therapeutically. The first investigation in which this potential has been explored is dry eye disease. In a series of studies in a mouse model, the topical administration of low μg/ml concentrations of CLU in a physiological buffer to the eyes was shown to provide complete protection from dry eye pathology and to rapidly resolve previously induced symptoms.^12^ Human clinical trials of CLU to treat dry eye pathology are being planned. Similarly, a recent study showed that: (1) CLU‐deficient mice were more susceptible to sepsis and endotoxemia, and (2) exogenously administered CLU bound to circulating histones to reduce their inflammatory, thrombotic and cytotoxic properties and improved survival in a mouse model of sepsis. This same study also reported that plasma CLU levels collapsed in human sepsis patients, and proposed that in pathologies with extensive cell death, CLU supplementation may improve disease tolerance and host survival.^13^ Inflammation is also strongly implicated in the pathology of Alzheimer's disease. Mutations in the *CLU* gene are one of the most significant risk factors for Alzheimer's disease, and multiple studies implicate CLU as protecting brain cells from toxic Aβ oligomers by neutralising their toxicity and mediating their safe clearance from the brain.^7^, ^14^

The brief outline above identifies several important disease scenarios in which natural CLU may prove to have high therapeutic value. As our understanding of the extracellular protein homeostasis system improves,^4^ one could expect more therapeutic opportunities to emerge. A promising direction exploits the possibility to rationally design functionally enhanced, engineered versions of CLU to provide even greater potency in specific therapeutic applications. CLU is a disulfide‐bonded glycoprotein with a relatively complex heterodimeric structure. This made it a challenging molecule to express recombinantly, let alone generate engineered mutants. However, the significant technical challenges were recently overcome, so that it is now possible to express and purify relatively large quantities of wild‐type and mutant CLU.^15^ move the following sentence to the start of the following paragraph CLU is a difficult candidate for analysis by X‐ray diffraction, cryo‐electron microscopy and nuclear magnetic resonance spectroscopy, and as a result there are currently no published structures available for full‐length CLU.

Nevertheless, the regions of CLU involved in specific interactions with misfolded proteins, histones, and cell receptors are under active investigation. Once these regions are identified, it will be possible to rationally design engineered versions of CLU with, for example, higher affinity binding to specific disease‐relevant ligands (e.g. Aβ, histones). It is already possible to design in silico complementary binding sequences for chosen epitopes in any target protein.^16^, ^17^ Therefore, by introducing such sequences into a molecular chaperone it becomes possible to enhance its ability to interact with a target protein. This strategy has been illustrated for the intracellular chaperone Hsp70, where an in silico‐designed binding sequence for α‐synuclein (a protein implicated in the causation of Parkinson's disease) was grafted onto the Hsp70 scaffold and significantly improved the ability of Hsp70 to inhibit α‐synuclein aggregation and toxicity.^18^

Other approaches to manipulate extracellular protein homeostasis for the purpose of disease therapy are under investigation.^4^ Therapeutic compounds could be used to: (1) enhance EC function, (2) upregulate EC secretion, (3) stabilise the native fold of extracellular proteins, (4) inhibit extracellular protein aggregation, (5) upregulate EC secretion, (6) enhance extracellular protease activity, or (7) promote cellular uptake for intracellular degradation.^4^ This list is likely to grow, as this is a new, almost completely untapped field, ripe with opportunities for bold thinkers. The translation of our growing understanding of extracellular protein homeostasis into effective new treatments for diseases associated with protein misfolding and aggregation, many of which are still intractable, is a goal with potentially huge future benefits for global health.
